# Deriving Flood-Mediated Connectivity between River Channels and Floodplains: Data-Driven Approaches

**DOI:** 10.1038/srep43239

**Published:** 2017-03-03

**Authors:** Tongtiegang Zhao, Quanxi Shao, Yongyong Zhang

**Affiliations:** 1 Commonwealth Scientific and Industrial Research Organization (CSIRO) Data61, Floreat, Western Australia, Australia; 2 CSIRO Land & Water, Clayton, Victoria, Australia; 3 Key Laboratory of Water Cycle and Related Land Surface Processes, Institute of Geographic Sciences and Natural Resources Research, Chinese Academy of Sciences, Beijing, 100101, China

## Abstract

The flood-mediated connectivity between river channels and floodplains plays a fundamental role in flood hazard mapping and exerts profound ecological effects. The classic nearest neighbor search (NNS) fails to derive this connectivity because of spatial heterogeneity and continuity. We develop two novel data-driven connectivity-deriving approaches, namely, progressive nearest neighbor search (PNNS) and progressive iterative nearest neighbor search (PiNNS). These approaches are illustrated through a case study in Northern Australia. First, PNNS and PiNNS are employed to identify flood pathways on floodplains through forward tracking. That is, progressive search is performed to associate newly inundated cells in each time step to previously inundated cells. In particular, iterations in PiNNS ensure that the connectivity is continuous – the connection between any two cells along the pathway is built through intermediate inundated cells. Second, inundated floodplain cells are collectively connected to river channel cells through backward tracing. Certain river channel sections are identified to connect to a large number of inundated floodplain cells. That is, the floodwater from these sections causes widespread floodplain inundation. Our proposed approaches take advantage of spatial–temporal data. They can be applied to achieve connectivity from hydro-dynamic and remote sensing data and assist in river basin planning and management.

Connectivity is an important concept in many disciplines, such as neuroscience^1^, ecology^2^, and environmental and earth sciences^3^. In hydrology, analysis of connectivity facilitates understandings of the dynamics of soil moisture and runoff at hillslope and watershed scales^4^,^5^. It also helps to investigate the ecological effects of hydrological processes^6^,^7^. However, a consensus on the definition and measurement of hydrological connectivity remains lacking^8^. For example, in hillslope hydrology, connectivity is defined as the physical coupling of discrete hydrological response units of the landscape by subsurface flow^9^. Meanwhile, in an ecological context, hydrological connectivity is conceptualized as the water-mediated transfer of matter, energy, and/or organisms within or between elements of the hydrologic cycle^10^.

Hydrological connectivity has static and dynamic aspects^5^,^8^. Structural connectivity is static and refers to the spatial distribution of landscape patterns that affect water transfer and flow paths; functional connectivity is dynamic and indicates how landscape patterns interact with rainfall input to produce runoff^11^. In general, the elements of structural connectivity can efficiently be described using hydrological indices, such as Euclidean distance and topographically defined hydrologic distance^4^,^12^. The elements of functional connectivity are more difficult to quantify than those of structural connectivity mainly because of their dynamic nature^5^. Therefore, functional connectivity is also called process-based connectivity because it is inherently presented in time-varying hydrological processes^8^.

The connectivity between river channels and floodplains plays a fundamental role in river basin planning and management^13^,^14^,^15^,^16^,^17^. In addition, the flood-mediated connectivity exhibits profound ecological importance because floods modify landscape and create heterogeneous habitats on floodplains^2^,^7^,^10^,^18^,^19^. This connectivity can be empirically derived using the criterion of Euclidean distance^20^. Specifically, a floodplain cell is connected to the nearest river channel cell, and its inundation status is inferred by comparing the relative elevation with the corresponding river stage^21^,^22^. However, apart from distance, other factors such as slope, vegetation, and surface roughness inevitably influence flood flow^23^. These influencing factors interact with each other and complicate the analysis of connectivity^3^,^6^,^24^. Hydrodynamic models formulate continuity equations of 2-D flood flow and parameterize the effects of different influencing factors^20^,^23^,^25^. While the flood-mediated connectivity is contained in model simulations, hydrodynamic models do not explicitly quantify the connectivity.

Recent advances in dynamic models have generated a vast amount of hydrological datasets^16^,^19^,^26^,^27^,^28^. For flood inundation, the dynamic process of floods travelling from river channels and propagating on floodplains can evidently be observed from temporal sequences of spatial simulation data^6^,^26^,^29^. However, methods to acquire connectivity from simulation data are generally lacking. Trigg *et al*.^26^ developed a novel statistical method to obtain connectivity from a time-series of spatial inundation data and illustrate the dynamics of connectivity during the flooding process. The statistical method measures how connected floodplains are, and our study elaborates on how floods connect river channels and floodplains. We extend the classic nearest neighbor search (NNS) to account for spatial heterogeneity and continuity. Further, we develop two novel data-driven connectivity-deriving methods, namely, progressive nearest neighbor search (PNNS) and progressive iterative nearest neighbor search (PiNNS). These approaches contribute to objectively deriving connectivity from the spatial and temporal characteristics of data. As will be illustrated later in this paper, PNNS and PiNNS are substantially better than NNS and effectively reveal flood pathways on floodplains and critical sections of river channels.

The remainder of the paper is structured as follows. First, a case study of floodplain inundation in the Flinders and Norman rivers, which are in Northern Australia, is introduced. Then, the NNS-, PNNS- and PiNNS-derived connectivities between river channels and floodplains are elaborated in the results section, followed by discussion and conclusions. In the last section, the mathematical formulations of the data-driven approaches are detailed.

## Case study

The data-driven connectivity-deriving approaches NNS, PNNS, and PiNNS are applied to the case study of the Flinders and Norman rivers in Northern Australia. The spatial–temporal dataset is generated by a 2D hydrodynamic model. The characteristics of spatial heterogeneity and continuity are illustrated on the basis of the simulated flooding process.

### Flinders and Norman Rivers

**Figure 1:**
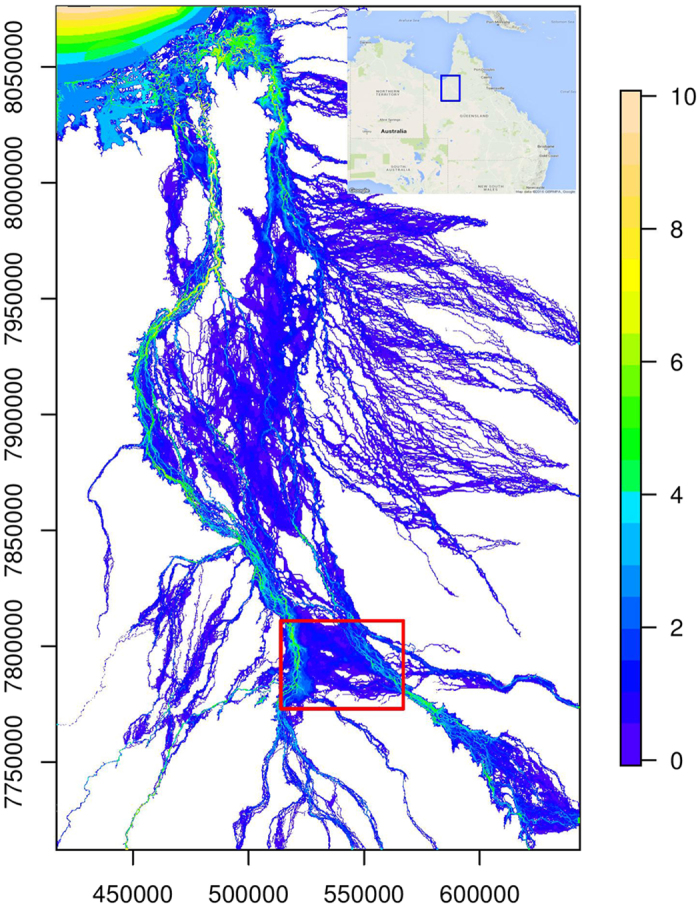
Flood extent map by MIKE 21 from the 1991 flood in the Flinders and Norman rivers. The red rectangle indicates the selected region where the flood-mediated connectivity between river channels and floodplains is detailed; the blue box in the inset map indicates the location of the study region. This figure is generated by the R-studio software version 0.99.465 (https://www.rstudio.com/), and the inset location map by the Google Maps module in R (https://cran.r-project.org/package=RgoogleMaps).

The Flinders and Norman rivers generally flow from south to north and into the Gulf of Carpentaria^30^. Flinders is the longest river in Queensland, with a length of 3,030 km and a drainage area of 111,163 km^2^. Norman River is on the east of the Flinders River; it has a length of 420 km and a drainage area of 50,665 km^2^. A defining characteristic of the two tropical river basins is the extensive coastal floodplain. The current land cover is mainly open savannahs and grasslands for cattle grazing. Although the region has considerable potential for agricultural development, considerable flood hazards exist^6^,^30^,^31^. The two river basins have a semi-arid tropical climate. The mean annual precipitation is approximately 500 mm, but more than 85% of precipitation falls during the wet season from November to April. Heavy rainfall caused by tropical cyclones results in widespread floods. Figure 1 illustrates the inundation extent and maximum inundation depth, as obtained from hydrodynamic simulation, from the 1991 flood. Evidently, a large part of the floodplain was inundated under the catastrophic flood. This study investigates the flood-mediated connectivity between river channels and floodplains. The connectivity is affected by river basin topography, which is pre-defined in hydrodynamic models as elevation and slope^23^,^25^. More importantly, it is subject to complex interactions between topography and floodwater from upstream catchments and sub-catchments within the study region^6^,^8^,^24^.

### Hydrodynamic simulation of the 1991 flood

In the Flinders and Gilbert Agricultural Resource Assessment project, a 2D hydrodynamic model MIKE 21 (DHI, 2009) was set up to analyze historical and future floods and to produce hazard maps^6^,^29^. The model domain is defined under the EPSG:28354 coordinate reference system. Coordinates of the south, north, west, and east boundaries are 416,965.3 m, 643,315.3 m, 7,712,109 m, and 8,076,159 m, respectively. The study region covers an area of 82,403 km^2^ and is represented by a raster DEM that contains 1,509 × 2,407 = 3,632,163 cells at a spatial resolution of 150 m × 150 m. A total of 196 sub-catchments are derived from the DEM. For each sub-catchment, the local flow is simulated using the Sacramento model; in the meantime, 11 flow boundaries contribute floodwater to the study region from upstream catchments^6^,^29^. Using the input flow data, the MIKE 21 model thus simulates unsteady flow in two horizontal directions in accordance with the basic principles of conservation of mass and momentum^23^.

**Figure 2:**
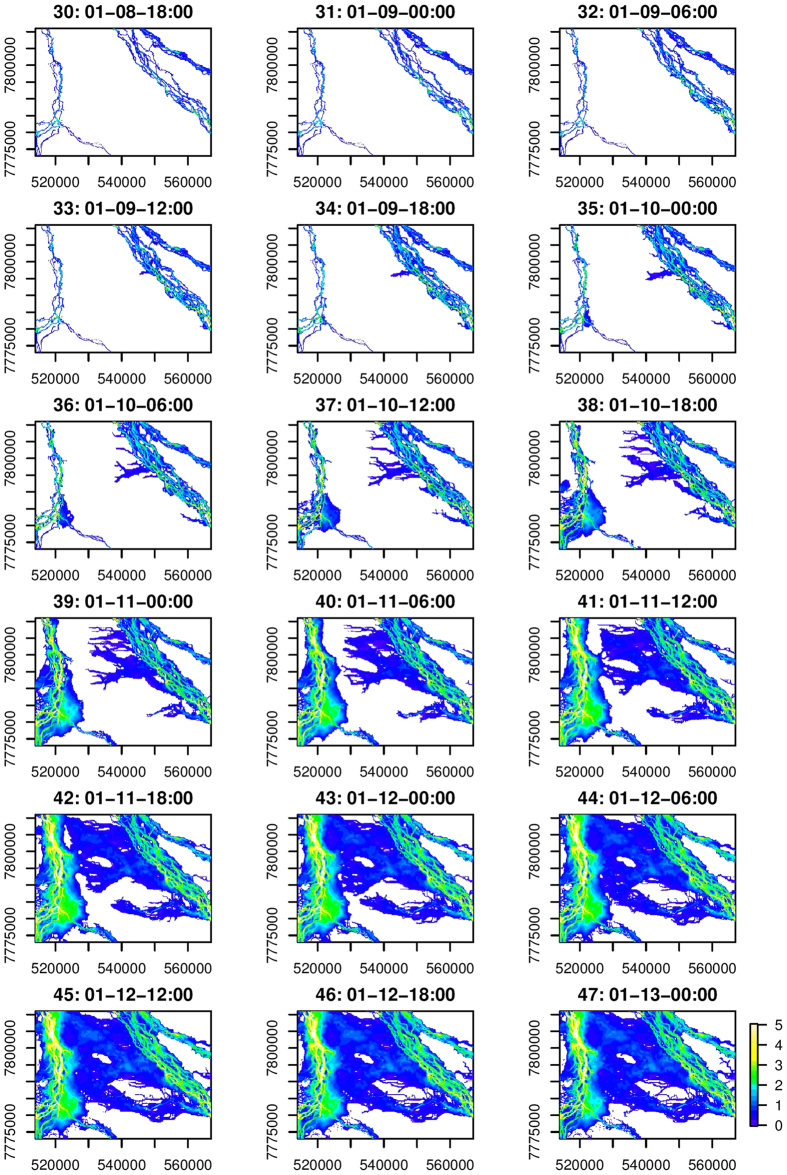
Progression of floodplain inundation and flood-mediated connectivity between river channels and floodplains. This figure is generated by the R-studio software version 0.99.465 (https://www.rstudio.com/).

The simulation of the 1991 flood is for the period from January 1^st^ 12:00 to January 23^rd^ 18:00. Raster files of the inundation extent and depth are saved from the hydrodynamic simulation every six hours and comprise 90 files. As the study region covers a large area, floods take several days to travel from upstream to downstream. Floodplain inundation along the upper reaches begins to recede, whereas floodplain in the lower reaches have yet to become inundated. Therefore, we account for the entire study region in the analysis, but we focus on a selected region (the red rectangle in Fig. 1) when presenting the results. Two major river channels are respectively located in the west and northeast parts of this region. This characteristic poses a substantial challenge. While the propagation of floodwater is clearly depicted in Fig. 2, associating the inundated floodplain with the two channels is not easy. Even in the simple case where only one river channel is present, several sections can possibly contribute floodwater to the floodplain.

### Properties of flood-mediated connectivity

The simulated flooding processes from January 8^th^ 18:00 (time step 30) to January 13^th^ 00:00 (time step 47) are detailed in Fig. 2. During the five-day period, the maximum inundation depth in the river channel rises from less than 2 m to nearly 5 m. In the meantime, the floodplain between the two river channels become inundated because of diffusive overbank floods. Floodwater connects river channels and floodplains. The dynamic flooding process indicates that the flood-mediated connectivity exhibits two important characteristics:

1. Spatial heterogeneity: an irregular expansion of floodplain inundation occurs. The inundated area along the west river channel gradually expands as time progresses. By contrast, floodwater from the northeast channel propagates along certain pathways and spreads on to the floodplains. In general, for a floodplain cell, a shorter distance to the river channel does not necessarily correspond to an earlier inundation.
2. Spatial continuity: the progression of floodplain inundation in each time step closely relates to inundated areas in the previous time step. This phenomenon reflects the fact that the “flood does not jump.” In other words, floodwater propagates gradually on the floodplain; it flows from areas that are already inundated to areas that are yet to become inundated.

Therefore, floods connect river channels and floodplains; the resulting connectivity is heterogeneous and continuous. These two characteristics are generally attributable to complex interactions between river basin characteristics and floodwater from upstream catchments and sub-catchments within the study region^8^,^23^,^28^. In this study, the proposed data-driven approaches (please refer to the Methods section for the details) aim to acquire the flood-mediated connectivity.

## Results

We apply PNNS and PiNNS, as well as NNS, to derive the connectivity between river channels and floodplains from the simulation data of the Flinders and Norman rivers. Inundated floodplain cells are connected to river channel cells. The connectivity analysis reveals flood pathways on floodplains and critical river channel sections.

### Flood pathways on floodplains

**Figure 3:**
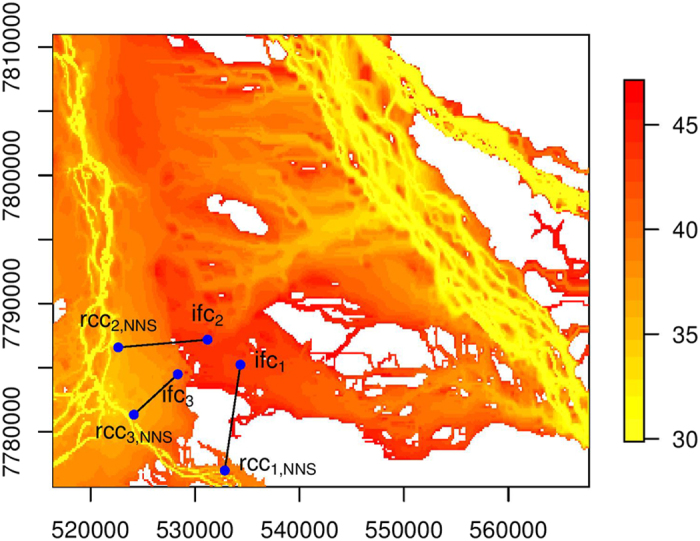
NNS-derived connectivity between selected floodplain cells and river channel cells. The color bar represents the time step at which the cells become inundated. This figure is generated by the R-studio software version 0.99.465 (https://www.rstudio.com/).

**Figure 4:**
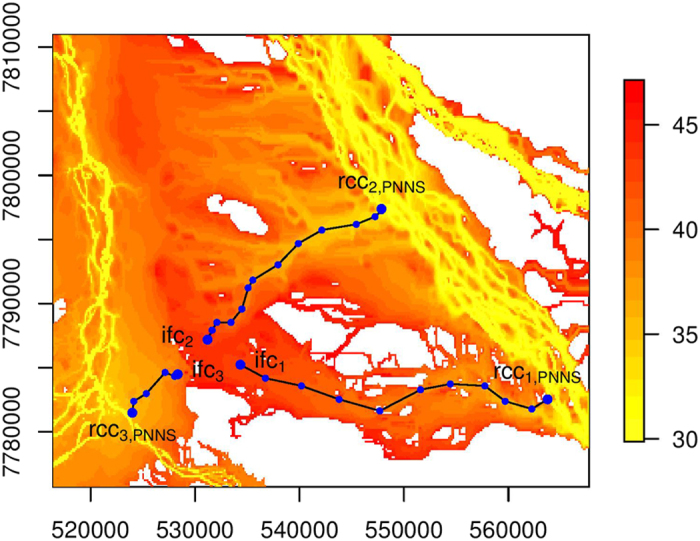
Similar to Fig. 3 but for PNNS. This figure is generated by the R-studio software version 0.99.465 (https://www.rstudio.com/).

**Figure 5:**
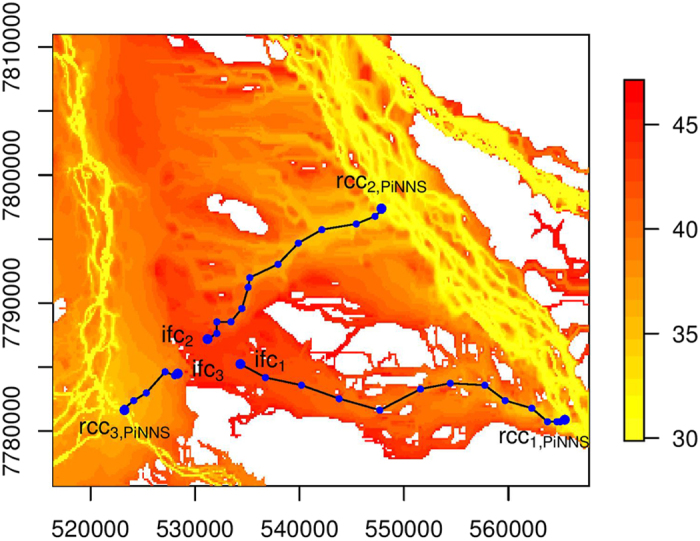
Similar to Fig. 3 but for PiNNS. This figure is generated by the R-studio software version 0.99.465 (https://www.rstudio.com/).

In the study region, there are two main river channels from which floodwater leads to floodplain inundation. We select three cells, ifc_1_, ifc_2_ and ifc_3_, from an area with a confluence of floodwater for illustration (Figs 3, 4 and 5). While these three cells are close to each other, floodwater that causes inundation at these cells can be observed to flow from different sections of the river channels (Fig. 2). Thus, the effectiveness of the data-driven approaches is tested through connecting these cells to river channels. Connectivity analysis is conducted from time step 31 to time step 47. Cells that are inundated before and at time step 30 constitute the set RCC of river channel cells. The results under NNS, PNNS, and PiNNS are presented in Figs 3, 4 and 5, respectively. In these figures, the time step when the cells become inundated is illustrated using a heat map. Yellower colors indicate earlier inundation, while redder colors later inundation. Thus, the difference in color represents the chronological order for the progression of floodplain inundation. The heat map shows certain branch-like structures that originate in particular from the northeast river channel and extend on the floodplain to the west channel. This pattern is associated with the progression of floodplain inundation (Fig. 2).

The NNS approach connects floodplain cells ifc_1_, ifc_2_, and ifc_3_ to river channel cells rcc_1,NNS_, rcc_2,NNS_, and rcc_3,NNS_, respectively. In Fig. 3, the connectivity is marked by solid straight lines. As expected, the connectivity by NNS is simply distance based. Unsurprisingly, the three selected cells are all connected to the nearby river channel in the west of the study region. However, the connectivity is noticeably not along the gradient of the heat map. In particular, the connectivity between ifc_1_ and rcc_1,NNS_ occurs over part of the floodplain that is not inundated. These results suggest that floodwater, which inundates the selected floodplain cell, may not be from the nearest river channel cell. In other words, although NNS finds the nearest river channel cell for each selected floodplain cell, there can be no physical relationship between the two cells.

The connectivity derived by PNNS is illustrated in Fig. 4. Cells ifc_1_, ifc_2_, and ifc_3_ are respectively connected to rcc_1,PNNS_, rcc_2,PNNS_, and rcc_3,PNNS_. The backward tracing for PNNS also identifies intermediate floodplain cells, which bridge the connectivity between the selected floodplain cell and the final river channel cell. As is illustrated, the connectivity in Fig. 4 is represented by solid polygonal lines that link a series of dots of intermediate cells. One major difference of Fig. 4 from Fig. 3 is that rcc_1,NNS_ and rcc_2,NNS_ belong to the river channel in the northeast of the study region. This result suggests that floodwater that inundates the two cells is not from the closer west channel but from the more distant northeast channel. This finding is confirmed by examining the polygonal lines against the heat map. It can be observed that the lines of connectivity tend to match the gradient of the heat map. This observation suggests that the connectivity by PNNS indicates the propagation of floodwater on the floodplain. As PNNS progressively applies NNS in each time step, it takes advantage of dynamically updated simulation data and facilitates more efficient connectivity analysis.

The connectivity by PiNNS is presented in Fig. 5. In some respects, the results are similar to those in Fig. 4. First, ifc_1_ and ifc_2_ are respectively connected to rcc_1,PiNNS_ and rcc_2,PiNNS_, which are located in the northeast channel, whereas ifc_3_ is associated with rcc_3,PiNNS_ in the west channel. Second, the connectivity is built through a series of inundated floodplain cells. The solid polygonal lines, which indicate connectivity, are along the gradient of the heat map as well. On the other hand, there are some subtle differences between PNNS- and PiNNS-derived connectivities. We check the coordinates of river channel cells and find that rcc_2,PiNNS_ is the same as rcc_2,PNNS_, but rcc_1,PiNNS_ and rcc_3,PiNNS_ are respectively different from rcc_1,PNNS_ and rcc_3,PNNS_. The differences are mainly due to PNNS not accounting for the spatial continuity in the connectivity analysis. A more in-depth comparison between PNNS and PiNNS is presented in the next section.

Figures 3, 4 and 5 highlight that the data-driven approaches PNNS and PiNNS are more effective than NNS in dealing with spatial heterogeneity and deriving the flood-mediated connectivity. We attribute their advantage to forward tracking and backward tracing. Forward tracking records how floodwater flows in each time step; then, backward tracing links pathways in individual time steps and illustrates how floodwater flows from a river channel cell to the selected floodplain cell. Compared with NNS, which directly connects an inundated floodplain cell to the nearest river channel cell, PNNS and PiNNS perform process-based analyses and illustrate flood pathways on floodplains. The pathways shown in Figs 4 and 5 essentially reflect flood-mediated connectivity.

### Critical river channel sections

**Figure 6:**
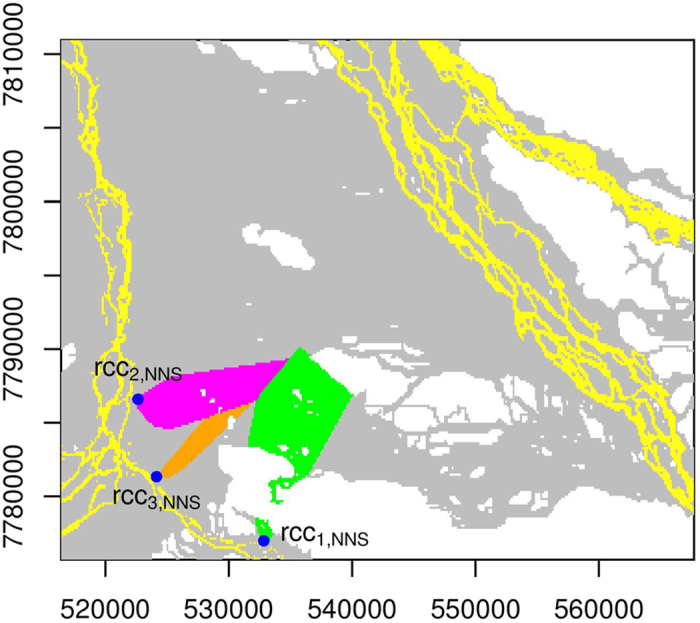
NNS-identified inundated floodplain cells that are connected to the three selected river channel cells. The sets of river channel cells and inundated floodplain cells are marked in yellow and grey, respectively; the three critical river channel cells are represented by blue dots, whereas the corresponding floodplain cells are marked in purple, orange, and green. This figure is generated by the R-studio software version 0.99.465 (https://www.rstudio.com/).

**Figure 7:**
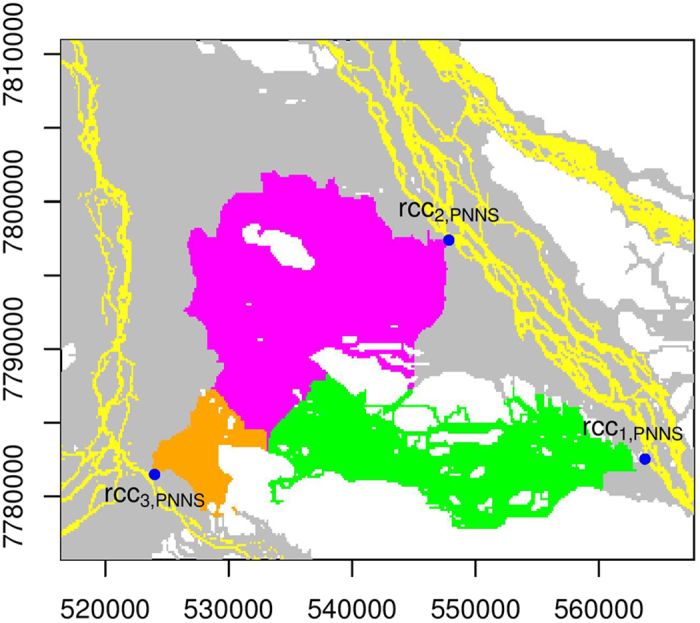
Similar to Fig. 6 but for PNNS. For the second and third river channel cells, some of their connecting floodplain cells cannot be continuously connected to them. For the first river channel cell, it is separated from the set of floodplain cells that are connected to it by PNNS. This figure is generated by the R-studio software version 0.99.465 (https://www.rstudio.com/).

**Figure 8:**
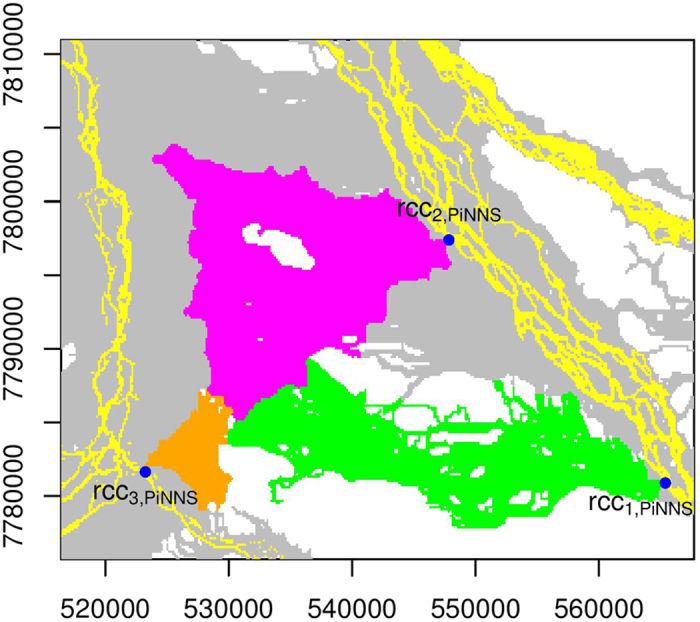
Similar to Fig. 6 but for PiNNS. This figure is generated by the R-studio software version 0.99.465 (https://www.rstudio.com/).

We perform connectivity analyses for all the inundated floodplain cells in IFC. Therefore, inundated floodplain cells that are being connected to a particular river channel cell are identified. NNS, PNNS, and PiNNS are respectively applied to analyze connecting cells for rcc_*i*,NNS_, rcc_*i*,PNNS_, and rcc_*i*,PiNNS_ (*i* = 1, 2, and 3). The results are presented in Figs 6, 7 and 8. Interestingly, each target river channel cell is connected to a number of inundated floodplain cells that form a set. In particular, larger sets are identified under PNNS and PiNNS. Given that the connectivities by PNNS and PiNNS relate to flood pathways, the results in Figs 7 and 8 suggest the existence of critical river channel sections. In other words, floodwater flowing from certain sections leads to widespread floodplain inundation.

The sets of floodplain cells connected to rcc_1,NNS_, rcc_2,NNS_, and rcc_3,NNS_ are obtained by NNS. Each river channel cell represents the nearest neighbor to its connecting floodplain cells. Figure 6 shows that this shortest-distance-based connectivity is actually not the flood-mediated connectivity. In particular, no continuous connection exists between rcc_1,NNS_ and most of the floodplain cells identified by NNS to be connected to it, indicating that floodwater cannot flow from rcc_1,NNS_ to these cells.

The PNNS approach identifies inundated floodplain cells for rcc_1,PNNS_, rcc_2,PNNS_, and rcc_3,PNNS_. Although Fig. 4 illustrates that PNNS tends to capture flood pathways, Fig. 7 suggests that certain limitations still hold for this approach. As for rcc_3,PNNS_, this cell does not continuously connect to all the floodplain cells that are identified to be connected to it. A similar observation applies to rcc_2,PNNS_. The worst case is for rcc_1,PNNS_. This cell is separated from the set of floodplain cells that are identified by PNNS to be connected to it. This separation can also somehow be observed from the pathway in Fig. 4. The polygonal line, which represents the connectivity between ifc_1_ and rcc_1,PNNS_, “jumps” over certain later inundated cells to rcc_1,PNNS_. An example of such a “jump” is further illustrated in the Methods section. Therefore, the step-by-step application of NNS in PNNS does not guarantee a spatially continuous connectivity.

The PiNNS approach devises iterative searches to ensure that the connectivity is continuous. For rcc_1,PiNNS_, rcc_2,PiNNS_, and rcc_3,PiNNS_, the connecting floodplain cells are presented in Fig. 8. Under PiNNS, the connection between rcc_*i*,PiNNS_ (*i* = 1, 2, 3) and the corresponding floodplain cells now exhibits spatial continuity, indicating that floodwater from each river channel cell can continuously flow to its connecting floodplain cells. The three sets of floodplain cells (Fig. 8) tend to match the branch-like structures in the heat map (Fig. 5). Overall, the results in Figs 6, 7 and 8 indicate that PiNNS performs the most effective connectivity analysis among the three approaches.

### Effects of elevation and river stage

**Figure 9:**
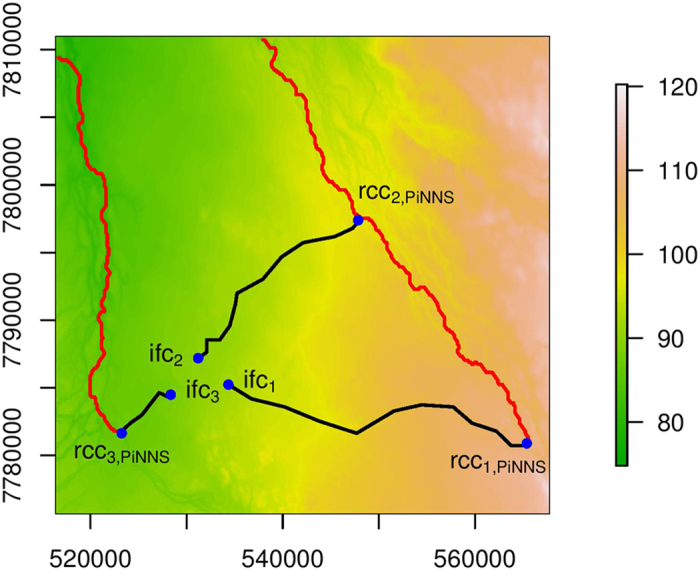
Illustration of the PiNNS-derived flood pathways (black lines) and the traditional flow paths in the direction of steepest descent (red lines) in an elevation map. This figure is generated by the R-studio software version 0.99.465 (https://www.rstudio.com/).

**Figure 10:**
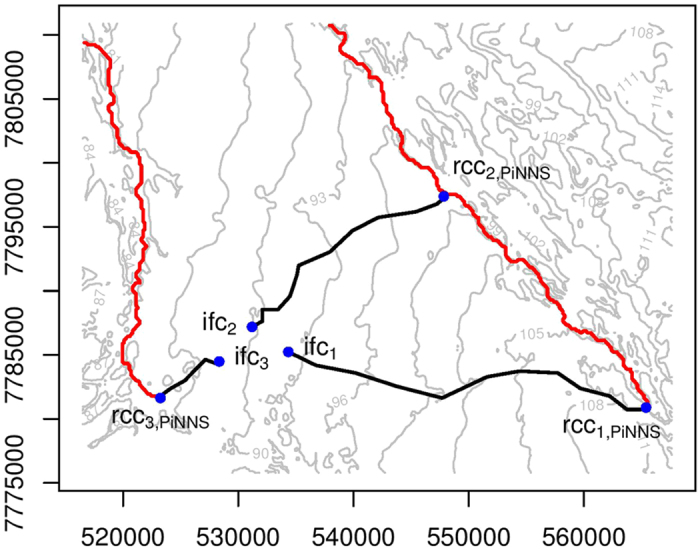
Similar to Fig. 9, but in a contour map. This figure is generated by the R-studio software version 0.99.465 (https://www.rstudio.com/).

The connectivity investigated in the two previous sections are mediated by the dynamic flooding processes that depend not only on basin characteristics, e.g., elevation and slope, but also on river stage^8^,^23^,^28^. While the effectiveness of PiNNS is demonstrated in the data-driven connectivity analysis, it is important to further validate the derived flood pathways and to associate the results with the physical influencing factors of flooding. For the region under investigation, we show the elevation in Fig. 9 and present the contour, from which slope can be inferred, in Fig. 10. The flood pathways from rcc_*i*,PiNNS_ to ifc_*i*_ (*i* = 1, 2, 3) by PiNNS are marked with black lines. As a comparison, flow paths from rcc_*i*,PiNNS_ (*i* = 1, 2, 3), which are obtained from elevation and slope in the direction of steepest descent^32^,^33^, are illustrated using red lines. It can be observed that the traditional flow paths in hydrology are different from the flood pathways by PiNNS. Regardless of the effect of river stage, flow paths are along river channels. In particular, the flow paths from rcc_1,PiNNS_ and rcc_2,PiNNS_ overlap. By contrast, subject to the effect of river stage, floods are no longer constrained in river channels and the pathways of overbank floods are much more diffusive (Fig. 2).

**Figure 11:**
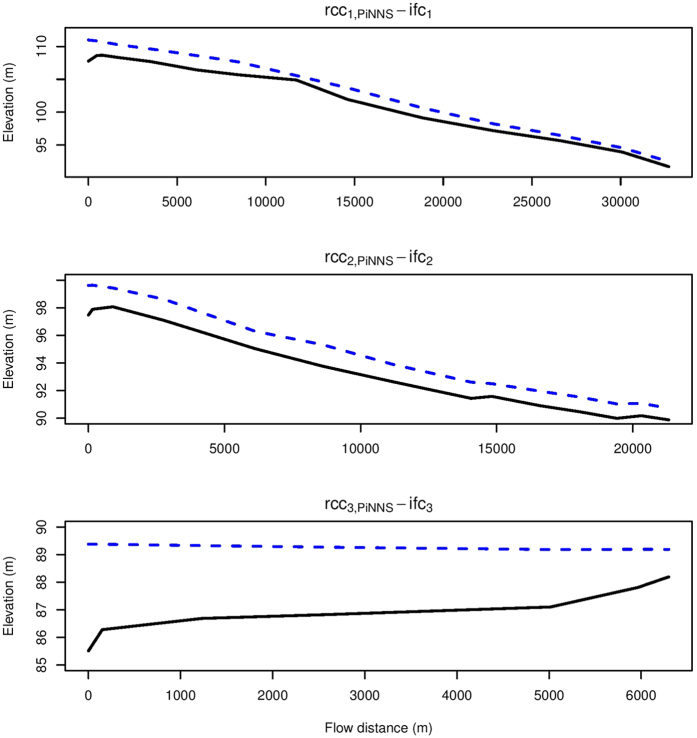
The elevation (black solid lines) and the maximum hydraulic head (the blue dashed line) along the PiNNS-derive flood pathways. This figure is generated by the R-studio software version 0.99.465 (https://www.rstudio.com/).

The elevation and hydraulic head along the PiNNS-derived flood pathways are examined in Fig. 11. In the three subplots, the x-axis represents the distance along the pathways from rcc_*i*,PiNNS_ to ifc_*i*_ (*i* = 1, 2, 3). The y-axis is for the elevation (the black soild line) and the maximum hydraulic head (the blue dashed line). The maximum hydraulic head, which is the sum of the elevation and the maximum inundation depth, generally decreases along the flood pathways. This pattern suggests that flood pathways derived by PiNNS are physically feasible. In contrast, the elevation does not show the same decreasing pattern. For example, the elevation from rcc_1,PiNNS_ (rcc_2,PiNNS_) to ifc_1_ (ifc_2_) increases slightly at the start and then decreases. Meanwhile, the elevation from rcc_3,PiNNS_ to ifc_3_ increases all the way. These increases in elevation suggest that water cannot automatically flow from rcc_*i*,PiNNS_ to ifc_*i*_ (*i* = 1, 2, 3). It also indicates that river stage plays an important part in the connectivity. Specifically, the river stage has to reach a certain threshold to facilitate the connectivity.

Another interesting finding from Figs 9, 10 and 11 is the role of relative elevation. In spatial analysis, relative elevation has been employed to infer inundation status and depth by comparing it to river stage^21^,^22^,^24^. This applies to ifc_3_. It can be observed that the maximum inundation depth at ifc_3_ is approximately the maximum river stage at rcc_3,PiNNS_ minus the relative elevation between rcc_3,PiNNS_ and ifc_3_. However, it does not apply to ifc_1_ and ifc_2_. The difference is mainly attributable to river basin topography. As shown in Figs 9 and 10, ifc_3_ is located in a river valley while ifc_1_ and ifc_2_ are on a hillslope.

## Discussion

The computational efficiencies of NNS, PNNS, and PiNNS are analyzed. A total of 3,632,163 cells are included in the case study, with RCC comprising 348,008 river channel cells (inundated before or in time step 30) and IFC including 614,441 inundated floodplain cells (inundated after time step 30). The classic NNS approach is computation-efficient^34^,^35^,^36^. It only needs 20.19 seconds to derive the connectivity between RCC and IFC on a Lenovo T410 laptop with an Intel Core i5 CPU (M560 2.67 GHz) and 4.00 GB RAM. Compared with NNS, PNNS and PiNNS entail further computation. The running times of PNNS and PiNNS are 131.33 and 181.39 seconds, respectively. Therefore, the progressive computation does not greatly increase the computation time. In particular, while PiNNS involves tedious iterations, it can also be deemed computation efficient. This is mainly because the set of cells whose connectivity has yet to be decided becomes smaller after each iteration. Given their high efficiency, the data-driven connectivity-deriving approaches have potential applications to case studies that are highly complicated.

Among the three approaches, PiNNS exhibits the most promising performance in connectivity analysis. One important output of this approach is flood pathways on floodplains. Important locations, e.g., residential areas, factories, and farms, are usually present on floodplains. PiNNS can readily be used to diagnose sources of flood hazards for these locations. By selecting the corresponding cells in the analysis, PiNNS not only reveals where the floodwater originates but also illustrates how it flows to selected cells. Another useful output of PiNNS is the identification of critical river channel sections. Floodwater from these sections causes widespread floodplain inundation. In river basin planning and management, flood defenses can be built around those critical sections. PiNNS can be used to analyze hydrodynamic simulation data before and after defense projects. Thus, the effects of such projects on floodplain inundation can be evaluated. In traditional flood hazard mapping, simulation data are examined empirically by experienced engineers. PiNNS can automatically extract useful information for flood hazard mapping and aid engineers in flood data analysis.

As the proposed approaches are driven by data, the derived connectivity reflects the spatial and temporal characteristics of data. For the connectivity to be reliably derived, it is important to ensure data quality. This involves a range of modelling issues, such as setting spatial resolution, choosing time step and formulating governing equations to represent the physical processes^23^,^28^. In this study, the connectivity analysis is built upon previous studies of hydrodynamic simulations^6^,^29^. When applying the data-driven approaches to other case studies, it would be important to first validate the hydrodynamic model and simulation data. This would lay the basis for an effective connectivity analysis. Further, it is pointed out that in hydrology, there are various types of connectivity that can be either static or dynamic^5^,^8^,^11^. The data-driven approaches in this study are particularly for the derivation of flood-mediated connectivity. This kind of connectivity is dynamic, and it exhibits spatial heterogeneity and continuity. We note that other approaches, e.g., statistical methods^26^, are available for the analysis of other connectivities.

## Conclusions

We develop PNNS and PiNNS based on the classic NNS to derive the flood-mediated connectivity between river channels and floodplains. Compared with NNS that derives a distance-based connectivity, PNNS accounts for spatial heterogeneity and incorporates dynamically updated simulation data into the analysis. PiNNS further considers spatial continuity. Both PNNS and PiNNS are used in forward tracking to derive the connection between newly and previously inundated cells. Then, through backward tracing, inundated floodplain cells are collectively connected to river channels. PNNS and PiNNS, as well as NNS, are applied to the case study of the Flinders and Norman rivers in Northern Australia. The results show that NNS-derived connectivity does not reflect the physical connection. PNNS improves and tends to capture the progression of floodplain inundation. PiNNS is the most promising because it devises iterative searches to ensure that the connectivity is derived continuously through adjacent cells. Among the three approaches, PiNNS is the most effective in identifying flood pathways on floodplains and critical sections of river channels.

Data–driven approaches take advantage of spatial–temporal data and extract connectivity from dynamic flooding processes. In this study, the utility of these approaches was demonstrated with simulation data. They can be further extended to other applications. For example, these methods can be readily used to exploit remote sensing data. Given their capacity to reveal flood pathways on floodplains and critical river channel sections efficiently, these approaches can serve as useful tools in river basin planning and management. Nevertheless, these data-driven approaches rely on data, and they cannot explain the mechanism that generates the data. Future studies can focus on elucidating the flooding mechanism and attributing the flood-mediated connectivity to the potential influencing factors, including river basin topography, floodwater, and hydraulic characteristics of floodplains and river channels. Insights into the mechanism are expected to facilitate physical understandings of flood-mediated connectivity. Such insights can also enhance the accessibility of PNNS and PiNNS and guide further improvements in the data-driven approaches.

## Methods

Spatial–temporal datasets from hydrodynamic simulation or remote sensing describe flooding processes in river channels and on floodplains. The data-driven approaches are meant to exploit the data and derive the flood-mediated connectivity between river channels and floodplains. PNNS and PiNNS are built on the classic NNS. PNNS accounts for spatial heterogeneity, whereas PiNNS further considers continuity.

### NNS

The NNS approach is a popular spatial analysis tool^20^,^34^,^37^. The shortest distance to the river channel, which is determined by NNS, is an important metric in floodplain delineation^21^,^24^. NNS facilitates a distance-based connectivity. That is, a floodplain cell is associated with the nearest river channel cell. The general optimization model for NNS can be formulated as follows:


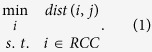


In Eq. (1), *dist*() represents an operator of the Euclidean distance between cell *i* and *j, i* is an element of the reference set *RCC* (the collection of river channel cells in our study), and *j* is the element for which the nearest neighbor is searched from *RCC*. Selecting *i* as the decision variable, the model determines the nearest neighbor i* in *I* for *j*.

The sets of river channel cells and inundated floodplain cells are denoted as *RCC* and *IFC*, respectively. The NNS optimization model that connects *IFC* to *RCC* is formulated as follows:


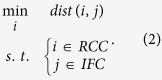


The NNS algorithm kd-tree^34^,^35^,^36^ is employed to solve Eq. (2). This algorithm is wrapped in an R package (https://cran.r-project.org/web/packages/RANN/index.html). One advantage of kd-tree is that instead of conducting one-by-one search (Eq. 1), it simultaneously determines the nearest neighbors, which are from *RCC*, for all elements of *IFC*, i.e.,


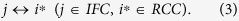


NNS facilitates straightforward connectivity analysis. However, when applying this approach to all the inundated floodplain cells, the obtained connectivity may, in many cases, fail to represent the physical connection between a river channel and a floodplain. This outcome is due to the fact that aside from distance, other factors such as vegetation, slope, and surface roughness influence flooding processes^6^,^23^,^24^. As a result, floodwater that inundates a certain floodplain cell may not be directly from the nearest river channel.

### PNNS

The PNNS approach takes advantage of the flexibility of NNS and applies NNS in a step-by-step manner. Specifically, NNS is implemented in each time step to identify the connection between newly inundated floodplain cells and previously inundated cells. The stepwise search in PNNS derives the connection progressively. Supposing the flooding process lasts for *T* time steps, the set of cells that become inundated in time step *t* is denoted as *G*_*t*_. In addition, supposing that flow remains in the river channel until *t*_0_ + 1, the joint set of *G*_*t*_ (*t* = 1, 2, …, *t*_0_) is taken as the set *RCC* of the river channel cells (Eq. 2):


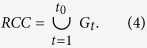


The joint set of *G*_*t*_ (*t* = *t*_0_ + 1, *t*_0_ + 2, …, *T*) is formulated as the set *IFC* of inundated floodplain cells:


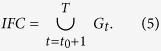


On the basis of hydrodynamic simulation data, the time step at which cells become inundated can be efficiently identified via Boolean operations. Considering that judging which cells comprise river channels and which cells constitute floodplains is not an easy task, this study separates *RCC* from *IFC* by time (Eq. 5) and thus circumvents the issue of river channel identification.

PNNS determines the connection for *G*_*t*_ (*t* = *t*_0_ + 1, *t*_0_ + 2, …, *T*), instead of the whole *IFC*:


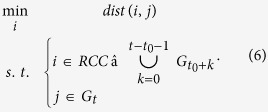


The connection between *G*_*t*_ (cells that become inundated at time step *t*) and 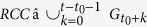 (cells that are inundated before *t*) is obtained as follows:


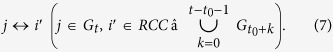


Stepwise search from time step *t*_0_ + 1 to *T* determines the connection for all the inundated floodplain cells.

**Figure 12:**
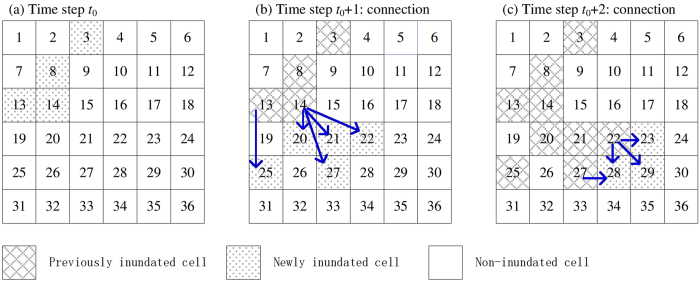
Schematic of PNNS (connections derived by NNS are marked by solid blue arrows).

The mathematical formulations of PNNS are illustrated in Fig. 12. A total of 36 cells are used; they are indexed by numbers 1 to 36, from left to right and from top to bottom. The indexing follows the arrangement of raster cells in spatial analysis:

1. At the initial step *t*_0_, the set of inundated cells is *RCC* = {3, 8, 13, 14} (Fig. 12a).
2. At time step *t*_0_ + 1, the connection between newly inundated cells 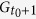 = {20, 21, 22, 25, 27} and previously inundated cells *RCC* = {3, 8, 13, 14} is determined by NNS (Fig. 12b):
  - Cell 14 is identified as the nearest neighbor for cells 20, 21, 22, and 27.
  - Cell 13 is identified as the nearest neighbor for cell 25.
3. At time step *t*_0_ + 2, NNS is re-applied to newly inundated cells 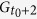 = {23, 28, 29} and previously inundated cells 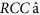 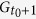 = {3, 8, 13, 14, 20, 21, 22, 25, 27} (Fig. 12c):
  - Cell 22 is the nearest neighbor for cells 23 and 29;
  - Two nearest neighbors are present, namely, cells 22 and 27, for cell 28.

Compared with NNS, PNNS accounts for dynamically updated simulation data in connectivity analysis. As a result, the PNNS-derived connection reflects the flooding process, particularly the spatially heterogeneous propagation of floodwater on floodplains. However, the applications of PNNS are limited by two issues. The first is that the connection may not be physically plausible under certain circumstances. As shown in Fig. 12b, cell 25 is connected to cell 13 based on the shortest distance, although cell 19, which is between these two cells, is not yet inundated. Thus, floodwater “jumps” from cell 13 to cell 25; the jump does not represent the actual flood pathway. This problem is handled using iterative searches in PiNNS in the next section. The second issue is that there can be more than one nearest neighbor (four at the maximum) for an inundated floodplain cell. For example, cell 28 is connected to cells 22 and 27. Without additional information, judging which cell is the “true” nearest neighbor is difficult. To account for relative elevation can help in this situation, but it introduces additional complexity to connectivity analysis and may fail to work because floodplains are usually flat. Considering this, the nearest neighbor is randomly selected following the default setting of kd-tree^34^,^35^,^36^.

### PiNNS

**Figure 13:**
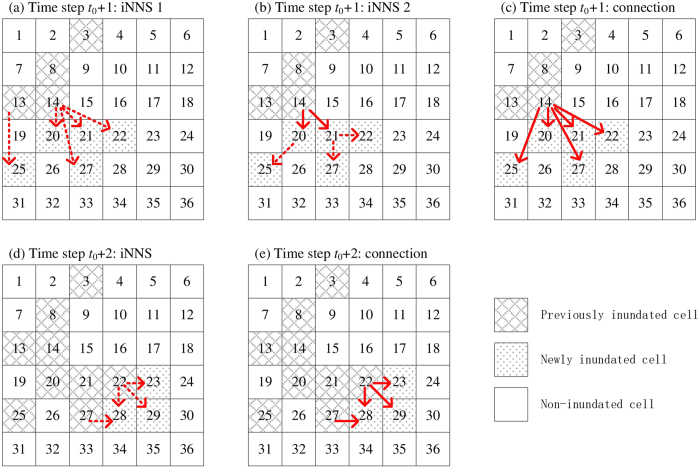
Schematic of iterative searches in PiNNS: dashed red arrows indicate iterative searches and solid red arrows represent iNNS-derived connection. (**a**) The first iNNS in time step *t*_0_ + 1; (**b**) the second iNNS in *t*_0_ + 1; (**c**) iNNS-derived connection between cells inundated in *t*_0_ + 1 and those inundated before *t*_0_ + 1; (**d**) the first iNNS in time step *t*_0_ + 2; (**e**) iNNS-derived connection between cells inundated in *t*_0_ + 2 and those inundated before *t*_0_ + 2.

The idea behind iterative searches in PiNNS is that “flood does not jump”. More specifically, floodwater that inundates one floodplain cell must flow over adjacent cells. Please refer to Fig. 13 for an illustrative example of the idea and the PiNNS approach. Eight adjacent cells correspond to one given cell. Supposing the size of cells is *d* × *d*, the distance to adjacent cells is either *d* or 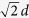. The minimum distance to non-adjacent cells is 2*d*. Considering that 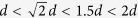, we choose a threshold at 1.5*d* (any value between 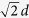 and 2*d* works) and devise iterative searches as follows:


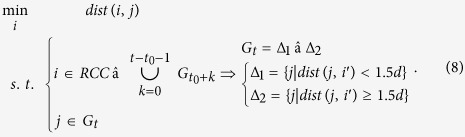


The left-hand side of Eq. (8) is the same as that of Eq. (6), and the right-hand side of the equation divides *G*_*t*_ into two subsets Δ_1_ and Δ_2_. Cells in Δ_1_ are adjacent to cells in 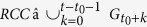, and their connection is built as follows:


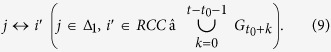


By contrast, cells in Δ_2_ are not neighboring 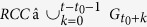. Their connection is determined iteratively as follows:


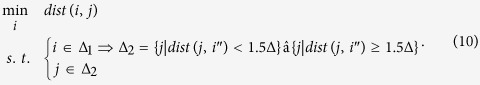


In Eq. (10), the nearest neighbor of *j* is denoted as *i*″. *i*″ ∈ Δ_1_ is differentiated from 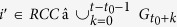 (Eqs 8 and 9). As illustrated on the right side of the equation, Δ_2_ can be further partitioned into two subsets by using the threshold distance. With regard to elements in the first subset 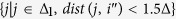, the connection is determined based on the connection of *i*″ as follows:


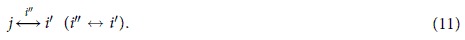


That is, *j* is connected to *i*’, to which *i*″ is connected. This operation results in a continuous connection between *j* and *i*’. The rationales are that *j* is adjacent to *i*″ and that *i*″ is continuously connected to *i*′.

For elements in the second subset 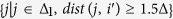, the connection is determined iteratively. At the beginning of each iteration, the two subsets on the right side of Eq. (10) are applied to update Δ_1_ and Δ_2_ on the left side of Eq. (10):


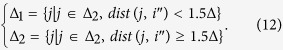


The iterative computations, namely, Eqs (10, 11, 12), progress until Δ_1_ becomes an empty set. In this situation, two outcomes are possible. One case is that Δ_2_ also becomes an empty set, which indicates that a spatially continuous connection has been derived for all the elements in *G*_*t*_ (Eq. 8). The other case is that Δ_2_ contains certain isolated cells for which the nearest neighbor cannot be obtained from adjacent cells. This phenomenon relates to noises in the simulation data. Another cause is that the time step at which the data are saved is slightly long. Floodwater propagates to and recedes from some cells within one time step. For these isolated cells, their nearest neighbors are identified from the other inundated cells in *G*_*t*_ and then connected to cells that are inundated before *t*.

The iterative applications of NNS in PiNNS are illustrated in Fig. 13 as follows:

1. In time step *t*_0_ + 1, the set 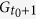 of newly inundated cells is {20, 21, 22, 25, 27}.
  - The first iteration determines the connection for 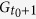 and partitions this set into Δ_1_ = {20, 21} and Δ_2_ = {22, 25, 27}. Cells 20 and 21 in Δ_1_ are adjacent to cell 14 and are connected to this cell (Fig. 13a).
  - The second iteration relates elements in Δ_2_ to elements in Δ_1_. Cells 22 and 27 are related to cell 21 and are finally connected to cell 14. Cell 25 is related to cell 21 and is thus connected to cell 14 (Fig. 13b).
  - Therefore, the connection for 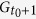 is derived by two iterative searches (Fig. 13c).
2. In time step *t*_0_ + 2, set 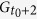 comprises {23, 28, 29} (Fig. 13d).
  - These three cells are all adjacent to previously inundated cells, with the distances being shorter than the threshold 1.5*d*. Thus, the connection is determined by one search (Fig. 13e).

PiNNS differs from PNNS in iterative searches, which ensures that a newly inundated cell is continuously connected to a previously inundated cell. As shown in Fig. 13, PiNNS connects cell 25 to cell 14. This connection is built through an inundated cell 20, which is also connected to cell 14 (Eq. 11). By contrast, PNNS connects cell 25 to cell 13. In this light, iterative searches in PiNNS are deemed to be more effective than the single search in PNNS. PNNS can obtain a connection that is inconsistent with the physical relationship in some cases, whereas PiNNS derives a spatially continuous connection between newly and previously inundated cells.

### Backward tracing for PNNS and PiNNS

A forward tracking process is used in PNNS and PiNNS. That is, in one time step, the connection is built between newly and previously inundated cells. These connections are pooled to form the connectivity between river channels and floodplains as follows:


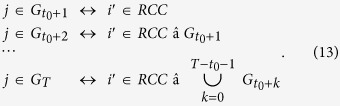


We devise backward tracing, which is essentially the inverse of forward tracking, to connect any *j* ∈ *IFC* to a river channel cell:

First, for the cell under investigation, the time step *t*_0_ + *k*_0_ (*k*_0_ > 0, based on Eq. 5) is identified when it becomes inundated. Using connections in Eq. (13), we connect this cell to another cell:


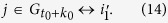


The period when 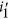 becomes inundated is denoted as *t*_0_ + *k*_1_. (*k*_1_ < *k*_0_, based on Eq. 13). Depending on *k*_1_, cell 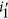 is from either *IFC* or *RCC* as follows:


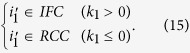


If 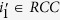, then *j* is successfully connected to a river channel cell. Otherwise, the connectivity of 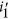 is analyzed as follows:


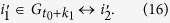


Similarly, the time step in which 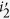 is checked to judge whether 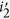 is a river channel cell. Therefore, an iterative process occurs as follows:


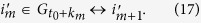


It progresses until 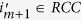. A series of inundated floodplain cells is identified in this process:


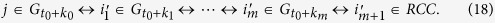


In Eq. (18), 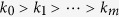. In consideration that flooding processes last from *t*_0_ to *T*, a floodplain cell needs, at the maximum, *T*–*t*_0_ time steps to become inundated. Consequently, an inundated floodplain cell can be connected to a river channel cell within *T*–*t*_0_ iterations.

**Figure 14:**
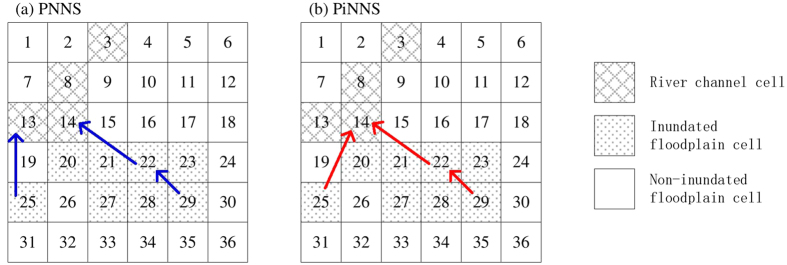
Schematic of backward tracing that connects inundated floodplain cells to river channel cells.

Two examples of backward tracing are demonstrated in Fig. 14: (1) Fig. 14a is for PNNS, and the backward tracing corresponds to the forward tracking in Fig. 12. Cell 25, which becomes inundated in time step *t*_0_ + 1, is connected to floodplain cell 13 in one iteration. Cell 29 is inundated at time step *t*_0_ + 2, and it is connected to cell 22 (inundated at time step *t*_0_ + 1) and then to floodplain cell 14. (2) Figure 14b is for PiNNS and relates to Fig. 13. Both cells 25 and 29 are connected to floodplain cell 14. With regard to PiNNS, all the inundated floodplain cells 20, 21, 22, 23, 25, 27, 28, and 29 are finally connected to cell 14 on the basis of the connections in Fig. 13c,e.

## Additional Information

**How to cite this article:** Zhao, T. *et al*. Deriving Flood-Mediated Connectivity between River Channels and Floodplains: Data-Driven Approaches. *Sci. Rep.* **7**, 43239; doi: 10.1038/srep43239 (2017).

**Publisher's note:** Springer Nature remains neutral with regard to jurisdictional claims in published maps and institutional affiliations.
